# XML-based approaches for the integration of heterogeneous bio-molecular data

**DOI:** 10.1186/1471-2105-10-S12-S7

**Published:** 2009-10-15

**Authors:** Marco Mesiti, Ernesto Jiménez-Ruiz, Ismael Sanz, Rafael Berlanga-Llavori, Paolo Perlasca, Giorgio Valentini, David Manset

**Affiliations:** 1 Università degli Studi di Milano. Via Comelico 39, 20135 Milano, Italy; 2 Universitat Jaume I. Avda. Vicent Sos Baynat S/N, E-12071, Castellón, Spain; 3 Maat Gknowledge, Méjico, 2. 45004 Toledo, Spain

## Abstract

**Background:**

The today's public database infrastructure spans a very large collection of heterogeneous biological data, opening new opportunities for molecular biology, bio-medical and bioinformatics research, but raising also new problems for their integration and computational processing.

**Results:**

In this paper we survey the most interesting and novel approaches for the representation, integration and management of different kinds of biological data by exploiting XML and the related recommendations and approaches. Moreover, we present new and interesting cutting edge approaches for the appropriate management of heterogeneous biological data represented through XML.

**Conclusion:**

XML has succeeded in the integration of heterogeneous biomolecular information, and has established itself as the syntactic glue for biological data sources. Nevertheless, a large variety of XML-based data formats have been proposed, thus resulting in a difficult effective integration of bioinformatics data schemes. The adoption of a few semantic-rich standard formats is urgent to achieve a seamless integration of the current biological resources.

## Introduction

Convergent advances in biochemistry techniques, biotechnologies, information technology and computer science provided the basis for the development of bioinformatics and made available huge and growing amounts of biological data [1].

Today's public database infrastructure spans a very large collection of heterogeneous biological data, opening new opportunities for molecular biology, bio-medical and bioinformatics research, but raising also new problems for their integration and computational processing. Indeed the integration of multiple data types is one of the main topics in bioinformatics and functional genomics, and several works showed that the integration of heterogeneous bio-molecular data sources can significantly improve the performances of data mining and computational methods for the inference of biological knowledge from the available data [2-5]. In this context a key issue is the representation of the basic bio-molecular entities and biological systems, their associated properties and data in a universal format interchangeable between different databases.

XML [6] has emerged as the most interesting recommendation for the representation and exchange of semi-structured information on the Web. The possibility to easily extend the structure and content of documents as well as the flexible association of schema information makes XML one of the main means for the representation of information exchanged on the Web and, in particular, of biological data. XML also provides a large set of other recommendations, standards and approaches that can be exploited for the representation and management of XML within database systems: query languages (like XPath and XQuery [7]) for querying collections of XML documents and obtaining adequate results; transformation facilities (XSLT [8]), for the presentation of the document contents with different formats (HTML, pdf, doc, etc.); description of schema information (DTD and XML Schema [9]) to enforce integrity constraints; SQL extension to handle at the same time (object-)relational and XML data (SQL/XML facilities [19]); indexing structures ([20]) for the efficient evaluation of queries. Moreover, many results from both the database and information retrieval communities have been presented for the integration and management of heterogeneous biological data represented through XML. Finally, new general purpose technologies (like Web Services, Grid computing, P2P data management systems) can be exploited to properly process heterogeneous bio-molecular data.

In this paper we first review the principal biological data types that have been identified and analyzed from the biological community and are currently available in different heterogeneous databases. Then, we present different proposals for the XML representation of many biological data types and the main initiatives that exploit XML for the integration of heterogeneous biological data. XML is thus not only employed for the exchange of data on the Web, but also for their management and integration. For what concerns data integration, we point out how conventional and advanced approaches based on Web services and P2P data management systems work specifically on XML and the key points and drawbacks of such approaches. Finally, we envision some future research directions for XML-based heterogeneous bio-molecular data integration, and also emphasize that further knowledge can be integrated with XML in order to overcome its limitations.

## Biological data types

In this section we introduce the main different types of bio-molecular data and their characteristics, considering also the database infrastructure that houses this information at different levels of representation.

### Primary sequence data

Historically the first types of data made publicly available have been nucleotide sequence data. It is well-known that *EMBL*, *GenBank *and *DDBJ *host primary sequence data with basic information about the sequence of DNA and RNA [21]. The content of these data bases (DBs) is the same as it constitutes the common base upon which most of the other bio-molecular DBs are built on. This integration effort is due to the international collaboration between the three most important bioinformatics institutions in Europe, USA, and Japan. Nevertheless, problems of accuracy and redundancy of the available entries of these databases can arise. These are due to both the quality of the annotations and biological representation issues (e.g. different Expressed Sequence Tags – EST – sequences are tissue specific and related to the functions of a specific gene). Thus, in some cases it would be necessary to identify such redundancies when dealing with multiple data sources.

Protein DBs represent the second important source of biological sequence data. The *SWISSPROT *DB is the reference protein bank for the "in silico" analysis of proteins and protein patterns, while *TREMBL *collects protein sequences obtained by translation from coding nucleotide sequences. Both the primary nucleotide DBs and SWISSPROT store sequence information in flat files, although an XML representation of these files is also available.

### Motif and domain data

Motifs and protein domains represent bio-molecular entities, usually discovered with pattern recognition methods applied to basic primary sequence data, which are widely used in bioinformatics and molecular biology research to characterize functions and families of proteins. Different specialized databases have been integrated in *InterPRO *[22], an EBI bioinformatics resource that allows the simultaneous search over different protein domain DBs, through *SRS *(Sequence Retrieval System) [23] or the Oracle DBMS. *Pfam *is a DB of families of proteins with common structural and functional elements [24]. They are represented through multiple sequence alignments and Hidden Markov Models. Entries are hierarchically structured from families, to domains, repeats and motifs. Pfam covers also families of proteins obtained through PSI-BLAST [25], an iterative version of the popular BLAST alignment tool for the progressive construction of profiles. The obtained multi-alignments and profiles are stored in the *ProDOM *DB [26]. Aminoacidic patterns, selected from protein sequences through experimental analysis and computational methods, are available in *PROSITE *[27]. Each entry of the DB is represented through a description of the pattern, bibliographic links, functional annotation and entries of the SWISSPROT DB where the pattern has been localized. The *PRINTS *DB represents families of proteins as a hierarchy, where families are related on the basis of their functionalities [28]. Each family is characterized by a "fingerprint", which is a set of *conserved motifs *deduced from multi-alignments.

### Structural data

Structural data of proteins refer to the atomic spatial coordinates of the atoms and aminoacids composing the protein itself. The reconstruction of the three-dimensional structure of a protein is of paramount importance to understand its function. Data are obtained by X-ray crystallography or NMR spectroscopy. Each entry of the PDB (Protein DataBase) is a file with several records and fields where all the details of the three-dimensional structure of the protein are available, as well as primary and secondary structure information and annotations [29].

### Gene level data

Although gene databases started with the annotation of primary sequence databases, recent advances in international projects for sequencing entire genomes have promoted the development of specific gene-centric data. For example, *Entrez Gene *provides a "gene-centered" view of bio-molecular data [30]. For each genetic locus, official gene names and synonyms, together with links to primary DBs are available.

All the information about the context of a specific gene are provided: information about transcripts, products, genomic regions, genotype, phenotype, related pathways and gene ontology terms are linked to the gene under investigation.

*KEGG GENES *is a collection of gene catalogs for all complete genomes and some partial genomes generated from publicly available resources [31].

This collection is part of *KEGG*, the Kyoto Encyclopedia of Genes and Genomes and provides a set of integrated databases that can be used to perform system level analyses [32]. KEGG GENES includes the KEGG Orthology (KO) system, a classification system of orthologous genes, including orthologous relationships of paralogous gene groups. Data about orthologous genes coding evolutionarily related proteins in different organisms as well as clusters of paralogous genes conserved in different species are available in *COG*: these data represent orthologs as clusters of individual proteins delineated by comparing protein sequences encoded in complete genomes [33].

Related DBs are represented by collections of nucleotide patterns with control and regulatory functions. For instance, *TRANSFAC *is a data bank for transcription factors involved in the regulation and activation of transcription [34]. Data refer to transcription factors and the corresponding DNA binding sites, and can be used for the analysis of gene regulatory events and networks. *UTRdb *is a database of the untranslated regions of eukaryotic transcripts [35]. They play a fundamental role in post-transcriptional processes of the regulation of gene expression, in the subcellular localization and translation of mRNA. Data related to both the post-translational modification and the regulation of translation are available in *TRANSTERM *[36].

### Genomic data

The characteristics and properties of bio-molecules can be investigated at the "omics" level: from the study and analysis of single genes or proteins the new bio-technologies introduced at the end of '90s permit to analyze the entire set of genes (genome) or proteins (proteome) of a given species. These data have been generated from the sequencing and mapping of the genome of entire organisms and are available as species-specific resources (e.g. *FlyBase *for *D. melanogaster *[37], *SGD *for *S. cerevisiae *[38], *MGD *for *M. musculus *[39]), or as integrated resources. For instance *Ensembl *collects data of the human genome and other organisms relative to gene mappings, functional annotations, transcripts, domains, mutations and other relevant information at genomic level [40]. Data are publicly available as flat files. Another similar genomic resource is represented by the *Genome Browser *[41].

### Transcriptomic data

DNA microarray data collect gene expression levels (i.e. levels of mRNA expressed in a given cell at a given time) at a genome-wide scale [42]. These data allow the analysis of the variability of gene expression between different tissues, individuals, or between different functional or pathological conditions. Three main projects developed at NCBI, EBI and Japan provide access to large collections of these data. *GEO*, Gene Expression Omnibus, provide structured data for platforms (probes that denote each spot on the array), samples (data of the molecules that need to be analyzed) and series (tables that link samples of an expression experiment to the corresponding platform). GEO is integrated within the NCBI Entrez web site [43]. *ArrayExpress*, developed at EBI is built on an Oracle DBMS, collects data MIAME-compliant (Minimum Information About a Microarray Experiment) using three main structures: Experiments, Array and Protocols. A subset of curated data can be queried on gene, sample, and experiment attributes [44].

### Polymorphism and mutation data

Polymorphisms and mutations data are now available in public databases and allow the analysis at genomic level of the associations between mutations and clinic phenotypes [45], as well as studies in the field of population genetics [46]. The database *dbSNPs *collects data relative to SNPs (Single Nucleotide Polymorphism), region polymorphisms and mutations associated to specific pathologies [47]. Other databases collect bio-medical data for the association between mutations and diseases. For instance *HGMD *(Human Gene Mutation Database) provides data obtained from literature about mutations and gene alterations related to hereditary diseases, with annotations that associate each mutation to the corresponding clinic phenotype. The *OMIM *(Online Mendelian Inheritance in Man) database reports data correlated to genetic Mendelian diseases. Data are collected in forms with phenotypes associated to chromosome alterations, to SNPs and mutations, with links to other databases (e.g. Entrez Gene) and cross-references to literature [48]. It is worth mentioning that *OMIM *provides an XML-based representation to export query results.

### System level relational data

The relationships and interactions between different entities and subsystems in cells at different levels (e.g. gene networks or the metabolism of an entire cell) represent a class of relational data by which we can model the behaviour of complex biological systems. These data, mainly obtained through high-throughput bio-technologies, can be used to infer the complex relationships between bio-molecules at "system level", considering biological phenomena as the result of the integration of different processes and different interactions involving the entire genome and proteome [49,50].

An example is represented by protein and genetic interaction data collected in *BioGRID *from major model organism species derived from both high-throughput studies and conventional focused studies [51]. BioGRID houses high-throughput two-hybrid [52] and mass spectrometric protein interaction data [53] and synthetic lethal genetic interactions obtained through synthetic genetic array and molecular barcode methods [54], as well as a vast collection of well-validated physical and genetic interactions from literature. Databases of biological networks offer other examples of relational data that can be used to model regulation processes of gene expression, and post-translational processes related to the metabolism and cellular transport of proteins. For instance the *KEGG PATHWAY *DB collects different interactions between proteins and genes represented through graphs: e.g. interactions between transcription factor and corresponding target genes, direct interactions (binds) between proteins, or relationship between enzymes participating to the same metabolic process. Other KEGG DBs are obtained by the systematic application of computational biology algorithms to the entire genome of an organism. For instance *SSDB *is a huge weighted, directed graph, where links corresponds to pairwise comparison of genes using Smith-Waterman similarity scores. The graph can be used to infer orthologs and paralogs or conserved gene clusters or as input to machine learning algorithms to predict gene functions.

## Advanced XML-based representations of biological data

The advent of XML as meta-language able to describe different kinds of data has led to the development of different XML-based languages for the description of biological data types.

In the last few years we have observed the proliferation of XML-based languages for the description of the (1) principal bio-molecular entities (DNA, RNA and proteins) and their structural properties, (2) gene expression (microarray), and (3) system biology. Initial proposals have been developed within small groups of institutes with the main aim of having a common representation of data structures and languages to model their own set of bio-molecular data types, whereas nowadays there are more initiatives (e.g. MIAME) to have a wider general agreement by specifying the minimal requirements that such kinds of data structures and languages should have. Table 1 summarizes some of the characteristics of a subset of existing XML languages (a further discussion on XML standards can be found in [55,56]).

**Table 1:** XML languages for the representation of biological data types

**Type of Data**	**Format**	**Concrete Scope**	**Version**	**Comments**
Molecular entities	BSML [57]	Biological sequences and sequence annotation	v.3.1/2005	Uses DTD. Included in EMBLxml.
	ProXML [58]	Protein sequences, structures and families	v.1.0/2006	Uses XSD. Included within HOBIT formats
	RNAML [59]	RNA sequence, structure and experimental data	v.1.1/2002	Uses XSD
	AGAVE [16]	Biological sequences and sequence annotation	2003	XSD Included in EMBLxml
	Uniprot XSD [121]	Representation of UniProt Records	2004	XSD, Successor of SP (SwissProt) ML format
	EMBLxml [17]	Biological sequences and sequence annotation	v.1.1./2007	Uses XSD. Currently includes BSML and AGAVE.
	GAME [18]	Genome and Sequence	v.0.3/1999	Uses DTD
	SequenceML	Sequence Information	v.2.1 2006	Designed to replace FASTA. Belongs to HOBIT XML formats.

Biological Expression	GeneXML [122]	Gene expression data	-	Uses DTD
	MAGE-ML [123]	Microarray expression data	v.1.0/2006	Uses DTD

System Biology	CellML [124]	Models of biochemical reaction networks	v.1.1/2006	Uses DTD. Available conversion to BioPAX.
	SBML [57]	Models of biochemical reaction networks	Lev. 2/2007	Uses XSD. Available conversion to BioPAX.
	PSI-MI [125]	Protein Interactions	v.2.5/2005	Uses XSD and OBO. Linked with OBO vocabularies.
	BioPAX [60]	Metabolic pathways, molecular interactions	Lev. 3/2008	Uses OWL. Linked to OBO vocabularies.
	CML [126]	Description of Molecules and Reactions	v.2.1./2003	Uses XSD

This table summarizes some of the characteristics of a subset of existing XML languages. In particular, we note the application scope, the number and year of the current version, and comments such as the kind of schema it relies on, or the interaction with other standards.

### XML representation of bio-molecular entities

The Bioinformatic Sequence Markup Language (BSML) [57] describes biological sequences (DNA, RNA, protein sequences) at different granularity levels via sequence data, and sequence annotation. A BSML document usually contains information about how genomes and sequences are encoded, retrieved and displayed. ProXML [58] is used to represent protein sequences, structures and families. A ProXML document consists of an identity section, containing the description of proteins, and a data section, containing properties of such proteins. RNAML [59] has been proposed for the representation and exchange of information about RNA sequences, and their secondary and tertiary structures. A RNAML document can represent RNA molecules as a sequence along with a set of structures that describe the RNA under various conditions or modelling experiments.

### XML representation of gene expression

The MAGE project [10] provides a standard for the representation of microarray expression data to facilitate their exchange among different data systems. MAGE mainly consists of: a data exchange model MAGE-OM (Object Model) and a data exchange format MAGE-ML (Markup Language) according to the standardization project groups responsible of the MIAME and MGED Ontology projects.

### XML representations for system biology

The need to capture the structure and content of bio-molecular and physiological systems lead to develop SBML (the System Biology Markup Language), CellML (the Cell Markup Language), BioPAX (the Biological Pathways Exchange Language) [60] and the set of HUPO-PSI (Proteomics Standards Initiative) formats [55]. SBML is used to encode models consisting of biochemical entities (species) linked by reactions to form biochemical networks, whereas, CellML encodes models consisting of a number of more generic components, each described in their own component elements. BioPAX and HUPO-PSI formats are examples of standards used to represent both structure and semantics of biological data. They are based on the use of ontologies as controlled vocabularies providing a non-ambiguous meaning of the domain.

### Integration initiatives

As showed above, several formats to represent biological data coming from different sources are available. Therefore, as a result, a large collection of heterogeneous biological data is available. This collection claims to be integrated to obtain a comprehensive view of the domain in order to perform analysis and sophisticated queries over the integrated data. *cPath *[61] has become an interesting initiative to use PSI-MI and BioPAX as standard exchange formats. cPath is an open software for collecting, storing and querying biological pathway data. Biological Databases can be imported and integrated into cPath via PSI-MI and BioPAX. cPath provides a standard web browser frontend and also a XML-based web service API in order to make data available to third-party applications for pathway visualization and analysis.

## Biological data integration

Biologists usually access different databases through their web interfaces, collect information (usually in text format) they think relevant and finally manually organize them in order to apply their algorithms and thus prove their theories. More and more there is the need to adopt (semi)-automatic approaches for the integration of biological data or rely on framework that help in the data integration process.

The integration of heterogeneous data sources is a traditional database research area whose purpose is to facilitate uniform access to a federation of several data sources. An integrated system provides its users with a global schema in which their views can be defined, along with the mechanisms needed to translate the elements of the global schema into the elements of the corresponding local schema, and vice versa. The heterogeneity of the integrated sources usually causes conflicts that must be resolved by the translation mechanisms in order to produce global results that are correct and complete.

**Figure 1:**
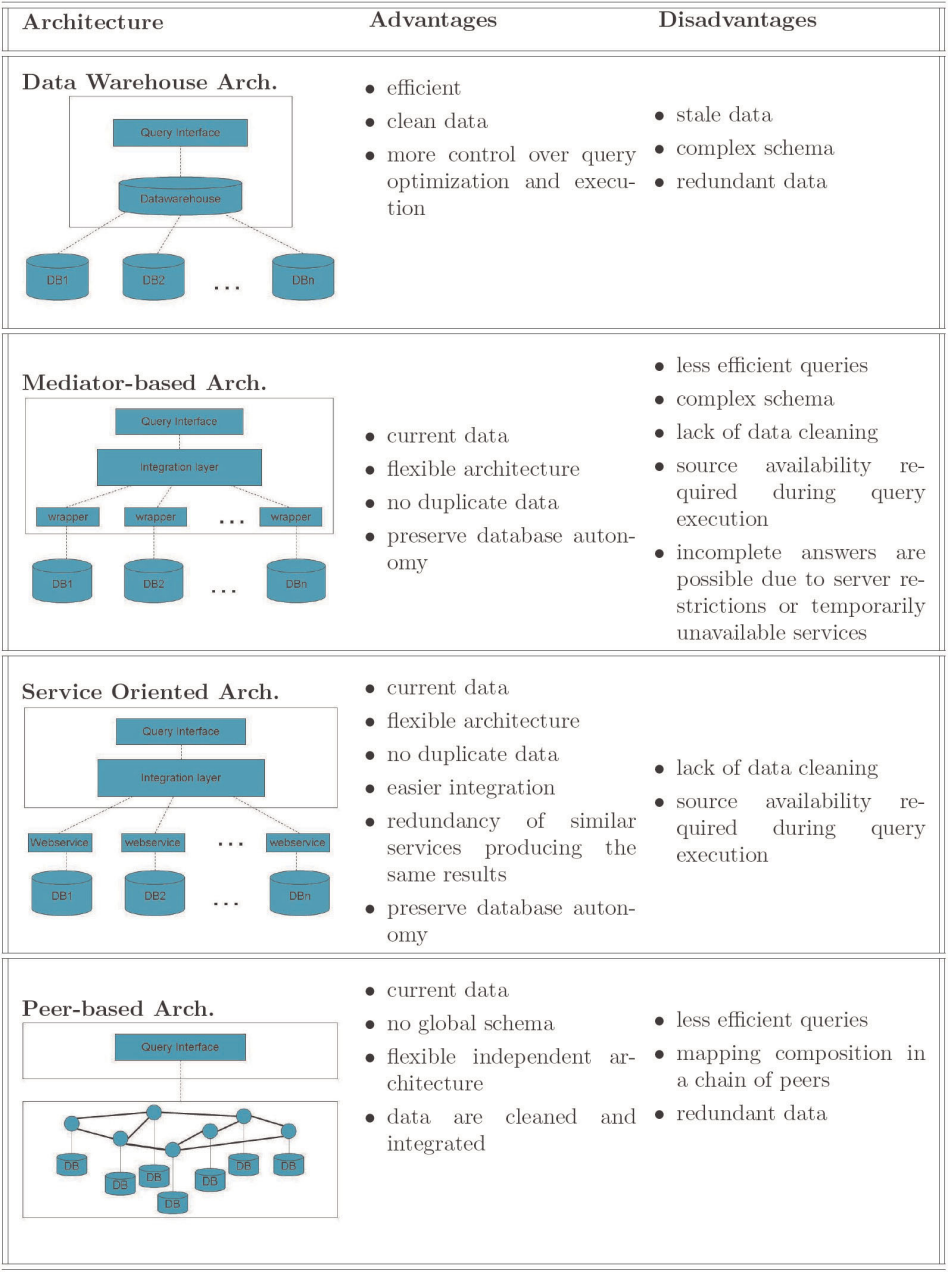
**Data integration architectures**.

Conflicts can be produced at different levels, namely: physical, syntactic and semantic levels. Currently, the adoption of Internet-based protocols and XML as interchange language has facilitated the integration at physical and syntactic levels. Indeed, XML technology has been formerly aimed at the syntactic integration through the definition of data models (DTD or XSD schemas), query languages (XPath and XQuery) and declarative transformation languages (XSLT). Additionally, recent XML-based formats like RDF and OWL also allow the specification of semantics for the objects to be integrated (ontologies). We remark that XML technology provides the languages for the representation of the information and lacks methods that implement the required integration. Data integration methods, formerly proposed in the database literature, are known as *integration architectures*. These architectures have been traditionally classified into three main groups: data warehouses, federated and mediated approaches (see Figure 1 for a summary of them).

In this section, we will analyse the combination of both XML and data integration architectures for biological data integration. Specifically, we start by introducing the aspects of comparison among the proposed data integration architectures. Then, for each type of architecture, we analyse how proposed systems address such aspects.

### Integration aspects

Table 2 summarizes the main dimensions we regard for comparing current approaches that integrates systems providing biological data. The next paragraphs are devoted to describe them and discuss their relevance.

**Table 2:** Summary of the integration aspects analyzed in this paper

**Aspect**	**Main approaches**
BioData	Sequences, Biological Expressions, Pathways, etc.
Instantiation	Materialized vs. Virtual integration
Integration	Common data storage, data access or data interface
Global View	Local As View, Global As View or Both As View
Global Model	Relational-based, Tree-based, Graph-based
Query Model	Ad-Hoc, SQL, XPath, XQuery, SPARQL, etc.
Semantics	Dictionaries, Thesauri or Domain Ontologies
Scalability	Low (*<*10 sources), Medium (20–50), High (*> *50)

This table represents the aspects around which biological data integration approaches are compared.

#### BioData

In this aspect we consider the kind of data to be integrated. Some previous papers like [62,63] have analysed the impact of data exchange formats in the integration of biological data and models. All formats rely on XML because of its simple syntax, extensibility and the numerous existing tools for its processing. Among the existing formats, SBML and BioPax are the most accepted ones for integration. As a result, a comprehensive list of converters are available from proprietary formats to SBML/BioPax as well as among themselves.

#### Instantiation

The degree of instantiation refers to where the physical data reside. In a virtual federation, data reside in the respective data sources, and the integration system gives a unified view of them, whereas in a materialized federation, data are collected from the data sources, cleaned, integrated and stored in a (physically) unique repository. Although the materialized approach is computationally more efficient, in general the virtual approach is chosen because it does not involve data replication, it is more flexible when further data sources should be included in the system, and it is easier to maintain [64].

#### Integration

The intended degree of integration is also a relevant aspect to take into account when comparing integrated systems. Thus, the integration architecture can be aimed at providing: 1) their common data storage, where biological data are homogenized and consolidated for end users, 2) their common data access, where all users can access (query) homogeneously all the integrated data sources and 3) their common data interface, where users build its tailored integrated applications by combining a series of components that share a common interface (e.g. web services).

#### Global View

**Figure 2:**
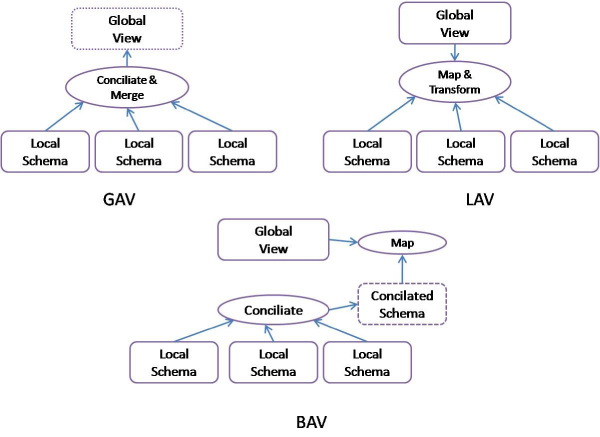
**Approaches to obtaining a global view**.

*Local As View *(LAV) means that the global model has been developed independently from local sources. Afterwards, local data is adapted to the global model in order to give a homogeneous and coherent data representation to end users. Instead, *Global As View *(GAV) means that the global model has been built by merging local source schemas, unifying entities at two possible levels: schema (S) and instance (I). Hybrid approaches (i.e. Both As View-BAV-) combine both aspects, there is a loosely defined global schema which is mapped to the set of reconciled local schemas (e.g. [65]). Figure 2 illustrates these three ways to generate an integrated global view.

#### Schema matching

One of the key issues for building a global view is the generation of mappings between local sources and the global view. In the literature, many approaches for automating the *schema matching *have been proposed [66]. Basically, a schema matcher is aimed at finding the possible mappings between the elements of two schemas. Such mappings are usually one-to-one but in many cases one-to-many mappings are required. One-to-many mappings are more complex to discover and require some transformation/operation to perform the integration (e.g. current and birth date in a schema must be subtracted to obtain the age in the other schema). Schema matching (SM) has been proposed formerly for relational schemas but it has been also applied to XML and OWL formats. For XML and OWL, SM also regards both the structural constraints and semantic constraints to validate the generated mappings. SM can be used in any of the three approaches: LAV, GAV and BAV. In LAV, SM maps each local source to the global view, in GAV is used to find the unifiable elements of the local sources and in BAV it is used for both.

**Figure 3:**
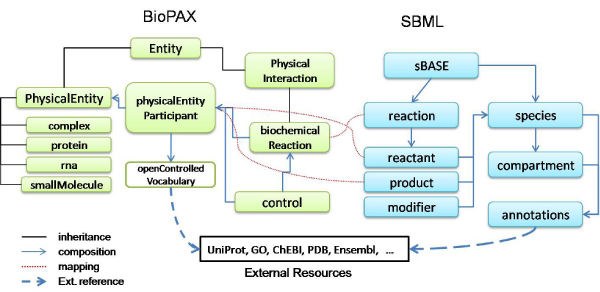
**Schema Matching example between BioPax and SBML formats**.

Regarding Biodata, the use of widely accepted formats like SMBL or BioPax greatly facilitates the generation of global views. SM is partially performed by a manual mapping between SMBL and BioPax (Figure 3). However, a true integration requires a deeper analysis of the values each data record contains. The integration at instance level is also facilitated by the use of external links to well-known resources such as UniProt, OMIM, GeneBank, HUGO, etc. In this case, the integration effort is focused in finding mappings between accession numbers and unique identifiers of these resources [67].

**Figure 4:**
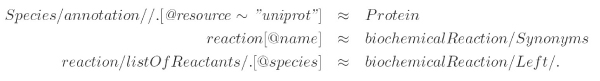
**Samples of mapping expressions**.

Following the schemas in Figure 3, Figure 4 shows examples of possible mappings. In these examples we have used XPath to locate the elements that participate in the mapping. Notice that the first rule involves two entities, the second one two entity attributes and the third one two entities by means of their context (reactants).

#### Global model and query language

The global model is the representation model for the unified local objects. The more expressive the global model is, the more complex is the global query processing. Traditional approaches rely on relational models (i.e. SQL) which are quite efficient. However, tree-like (e.g. XML) and graph-like (e.g. RDF) models are much more adequate for representing most biological data. The counterpart is that these models present a higher complexity for query processing (e.g. XQuery and SPARQL query processors).

#### Semantics

Ontologies have been used as mediator schemas defining an abstract layer (semantic level), away from data structures and implementation strategies (physical level), in order to provide a transparent access to heterogeneous resources. Gruber [68] defined ontology as an "explicit specification of a conceptualization". An ontology specifies the concepts and relationships (vocabulary) which are relevant for modelling a domain, moreover it provides a meaning for that vocabulary by means of formal constraints. This definition is rather broad and the concept ontology is not always exploited as desired. Instead, thesauri and glossaries, which have less logical expressivity, are used to facilitate data sources interoperability and integration, that is, which terms of the sources are intended to have the same meaning. Further discussion of the advantages of expressive ontologies are given in Section "*Towards more powerful representations of bio-entities*".

#### Scalability

An integrated systems is said to be scalable if the cost of adding new participants (e.g. sources or components) to the integrated system is low. This cost will mainly depend on the difficulty of updating the global view.

### Data warehouse approaches

A data warehouse integrates and aggregates data of several different DBMSs into a single repository. To this end an integrated database schema is developed that encompasses the schemas of the sources to be integrated. Moreover, views targeted to the analysis to be performed can be realized. Usually an integrated database schema is developed from scratch and can be seldom updated. Updates should be performed sparingly even if, due to a change of user requirements, they are mandatory.

Systems that rely on the data warehouse architecture are usually restricted to consider a few source databases, but can achieve a higher degree of integration of the data sources. The limitation of warehouse system is mainly due to the difficulty to integrate in the system new data sources without changing the schema of the data warehouse. Therefore, these systems allow to obtain an high degree of instantiation. Examples of these systems are the following ones:

• DWARF [69], which integrates data on sequence, structure, and functional annotation for protein fold families. DWARF extracts data from public available resouces (e.g. GenBank, ExPDB and DSSP).

• BioWarehouse [70] is an open source toolkit for constructing bioinformatics database warehouses by integrating a set of different biological databases into a single physical DBMS (MySQL or Oracle). It supports data related to the following types of biological objects: genes and genomes, proteins, enzymatic reactions, biological pathways, taxonomies, nomenclatures, microarray gene expression, computationally-generated results.

• Atlas [71] locally stores and integrates biological sequences, molecular interactions, homology information, functional annotations of genes, and biological ontologies.

• Biozone [72,73] is a unified biological resource on DNA sequences, proteins, complexes and cellular pathways. Biozon combines graph model and hierarchical class approaches to express and characterize biological entities in terms of constraints depending on the relations with other modelled entities or depending on the proper nature of each individual entity. Biozon supports derived data strategies based on similarity relationships and functional predictions enabling propagation of knowledge and allowing the specification of complex queries.

• cPath [61] is an open source database software for collecting, storing and querying biological pathway data. Multiple databases can be imported and integrated into cPath via PSI-MI and BioPAX standard exchange formats. cPath data can be viewed by means of a standard web browser or exported via an XML-based web service API, making cPath data available to third-party applications for pathway visualization and analysis.

Most of these approaches take the LAV strategy to build the global view, and provide a common data and storage model (see Table 3). Due to the complexity of the data loaders, where transformations between schemas are usually hard coded (e.g. Java, C++ and Perl programs), the cost of adding new sources is high. This problem can be alleviated if data sources already provide their data in standard XML formats, in which case a few data loaders (e.g. a BioPax data loader) can deal with many sources. However, any evolution in either the exchange formats or the source schemas will imply a re-implementation of all these loaders, so the cost of maintaining these integrated systems can be very high.

**Table 3:** Data warehouse approaches

**Aspect**	**DWARF**	**BioWareh**.	**Atlas**	**Biozone**	**CPath**
BioData	Sequences	All Types	Genes	All Types	AllTypes
Instantiation	Materialized				
Integration	Common Storage/Access				
Global View	LAV			GAV (I)	LAV
Global Model	Relational			Graph	RDF/OWL
Query Model	SQL			SQL/AdHoc	SPARQL
Semantics	-	Thesaurus	-	-	Ontologies
Scalability	Low	Medium	Medium	Medium	Medium

This table compares the datawarehouse approaches relying on the aspects introduced in Table 2.

### Mediation approaches

In contrast with data warehouse-based architectures, in mediator-based systems (originally proposed by Gio Wiederhold [74]) individual data sources maintain their independence. Data integration is achieved by defining a *global view*, or integrated schema, which is shared by all sources; a "mediator" component, or mediator-based middleware, adapts queries formulated against the global view to the local data and capabilities. Typically, each individual source will also require the definition of a "wrapper" component, which will be used to export a view of the local data in a useful format for mediation (by translating the data to/from XML, for instance). Figure 1 depicts a typical mediator-based architecture. Query processing is achieved by sending subqueries to relevant sources, and then combining the local query results.

Thus, the main advantages of mediator-based architectures are threefold: (*i*) the insurance that returned data are always up-to-date, since queries are performed dynamically (*ii*) data are not duplicated since they reside in their local repository, (*iii*) it is easier to add new sources of information. A major drawback of mediator-based system is the need to manually specify the mappings between local and global schemas; several techniques have been proposed to automate these steps (e.g. [66]).

The following systems are examples of mediator-based systems for biological data:

• Ontofusion [75] proposes a multiple ontology approach to integrate genomic and clinical databases at the semantic level. For each data source an ontology (named virtual schema) is created to describe the structure of the data. Virtual schemas are unified (i.e. merged) in a unique global schema to give an homogeneous access to data.

• TAMBIS [76], unlike Ontofusion, adopts a unique ontology approach to provide a common access to several data resources so that cross database searches seem to be transparent. An ontology called TAO (Tambis Ontology) has been created for this purpose. TAO collects all the requirements of the database to be integrated. Scalability, when adding new resources, is the major drawback of this approach.

• BioMediator [77] uses a logic-oriented knowledge base to store meta-information about each data source, which allows the specification of tailored mediated schemas including rich relationships. The mediator component is extensible through the use of *plug-ins*, which allows the definition of mapping rules for the tailored schema.

Table 4 summarizes the main mediator-based approaches. Last two columns report the characteristics of two recent internet-based architectures that facilitates the integration of systems: Web Services and Peer-to-Peer architectures. Both architectures are discussed in the next sections.

**Table 4:** Mediator-based Approaches

**Aspect**	**Ontofusion**	**TAMBIS**	**Biomed**.	**WS**	**P2P**
BioData	Genes	All types	Genes	All Types	All Types
Instantiation	Virtual				
Integration	Common Access				
Global View	GAV (S/I)	GAV (S)	LAV	LAV	N.A.
Global Model	RDF/OWL		XML	RDF/OWL	XML
Query Model	Boolean	CPL	XQuery	SPARQL	XQuery
Semantics	Ontologies		-	-	-
Scalability	Medium	Low	Medium	High	High

This table compares the mediator-based approaches relying on the aspects introduced in Table 2.

(Biomed. = Biomediator, WS = Web Services approaches, P2P = peer-to-peer approaches).

In general, in the Bioinformatics area, mediator-based approaches are less popular than data warehouse ones. One possible reason for this is that mediator-approaches require reversible transformations in order to both distribute global queries to local sources and translate local results as global objects. Data warehouse approaches only require unidirectional transformations (i.e. from local to global view), which makes their implementation easier.

### Service-oriented architectures (SOAs)

In the previous sections we have been mainly concerned with the integration of biological data sources through the classical data warehouse and mediator approaches. However, Bioinformatics research usually implies processing all these data by means of software applications as those that realize *in silico *experiments. In this context, Service-Oriented Architecture (SOA) provides a standard method to integrate both data sources and software applications by regarding them as interoperable *services*. Thus, client applications will combine these services to implement their intended tasks. In this section, we review the main efforts in providing such services within the Bioinformatics community.

**Figure 5:**
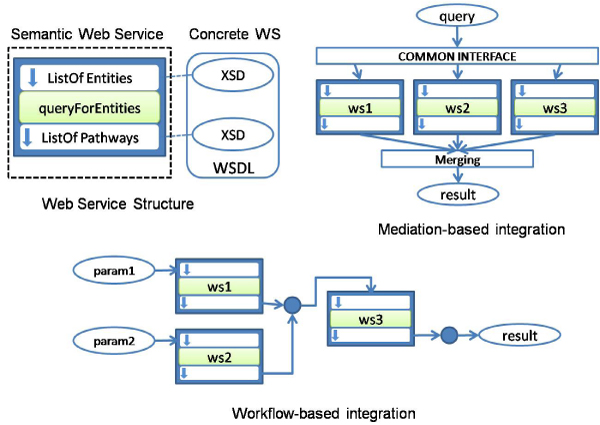
**Integration through Web Services**.

Figure 5 shows an *abstract *Web Service (WS) for retrieving pathways given a set of possible participants. It is represented with a box with three parts: the input, the method name and the output or result. This web service can take part of either a mediator-based architecture (top right part of the figure) or a workflow (bottom part of the figure). However, in order to use *concrete *web services (i.e. web services located at some machines with a specific interface), applications and users must be aware of the XML schema of input and output parameters. This schema is expressed with the Web Service Description Language (WSDL). Thus, the main integration issue consists of reconciling the schemas of the services to be combined. Biological research institutions like the National Center for Biotechnology Information (NCBI) and the European Institute for Bioinformatics (EBI) have published most of their applications and data sources as Web Services. Thus, researchers can freely invoke the Entrez e-utilities, the EMBOSS suite [11], the EMBL-EBI tools [12] and Distributed Annotation System [13] among others. These Web Services constitute the basic layer over which more complex services and workflows can be defined.

#### Semantic web services

WSDL files have found very limited usage for processing and distributing biological data. As a consequence, new protocols have been proposed to extend the basic functionalities of bioinformatics Web Services. BioMOBY [78] has been quite successful as such an extension. MOBY services are registered in a central node by properly annotating their interfaces. Such annotations mainly involve the input and output data of each service as well as some descriptions about its functionalities. Currently, there are more than 1000 services registered and more than 500 data types associated to their descriptions (see ). Notice that the ratio between data types and services indicates that a further data integration effort should be done in order to make them more interoperable.

#### Workflows

Several proposals have recently appeared to define complex workflows over BioMOBY services to perform for example *in silico *experiments. The most popular of these proposals is the Taverna tool [79], which has been proposed within the myGRID project [80]. This tool allows users to first define graphically a workflow (i.e. chain of service invocations) and then execute it over a GRID-based middleware. Other similar Web-based tools have been proposed, for example MOWServ [81], SeaHawk [82] and Remora [83] to mention a few. Recently, some extensions to the BioMOBY protocol have been proposed according to the new requirements arisen from workflow management [84].

#### Grid-based services

Grid technologies are intended to provide highly scalable computing frameworks where resource-hungry applications can be performed efficiently. As the biological community is continuously generating vast amounts of biological data, which also require time-consuming processes to be analyzed, Grid computing has been usually taken up in large bioinformatic projects (e.g. myGRID, caBIG, EGEE, etc.) Grid technologies also rely on Service-Oriented Architectures. Indeed, recent standards for Grid architectures basically extend the Web Service technology. Thus, the Web Service Resource Framework (WSRF) is the WS extension proposed for the Open Grid Service Architecture (OGSA). Unlike Web Services, Grid services must account for security, transaction and distribution issues arisen from Grid architectures. A good review of Grid technologies applied to Bioinformatics can be found in [85].

Service-Oriented Architectures have an increasingly prominent role in the development of biologiocal data processing and integration. As a result, SOAs are constituting the technological basis for almost the projects aimed at seamlessly integrating biological information systems. Nevertheless, little work has been done in developing specific methods for querying homogeneously biological data-providers services.

### Peer architecture

All the previous presented architectures rely on the definition of a global schema that is well accepted by all data sources belonging to the integrated systems. Current efforts are devoted to the definition of peer networks where data can be locally organized and managed [86]. Each peer or group of peers can share the same schema, and local mapping among pairs of schemas can be established leading to the formulation of a semantic network. When a new peer wishes to join the semantic network, it should establish a mapping simply with a single peer or a subset of the network peers. When a query is submitted to one of the network peers, the query is routed to the peers that, according to the resource availability policies, can contain possible answers. Relying on the pre-established mappings among schemas, it is possible to translate a query to be executed in local schema (and thus obtain more precise results) or to translate the results in order to make homogeneous and comparable the different results. The peer, that initially received the query, is in charge of collecting the answers and returning them to the requesting user or application. Key features of a peer architecture is, thus, the lack of a global huge schema. Peers can develop schemas that are tailored for their main users and then establish a mapping with a small fraction of other peers. A peer can easily join and leave the network. The main drawback of this architecture is the need to develop mappings and their use on the fly to evaluate queries that can effect the performance of the retrieval process. Many efforts are currently devoted to quickly perform these tasks (e.g. developing mapping tables [87]). As in the other architecture, XML plays a central role in semantic peer networks, XML can be exploited both as a message exchange format among peers as well as a format for the representation of the peer contents.

Well-known and general purpose P2P data management systems (PDMS) like Hyperion [86], PeerDB [88], and GridVine [89] have been proposed that rely on the relational model and can be exploited for the management of biological data that do not present complex structures. Moreover, the Bioscout system [90] has been developed for helping biologists in the graphical specification of queries and for developing efficient query plans to be executed in a peer network. Apart from these few systems, P2P technology has been scarcely applied to the biological research.

From a practical point of view, there are not big differences between Service-Oriented Architectures (SOA) and P2P. Both have as strongest point their good scalability. However, unlike SOAs, P2P systems lack a solid and standard technological background (e.g. SOAP, WSLD, OGSA etc.) that makes them fully interoperable.

## Advanced issues in XML-based biological data integration

Even if several XML-based approaches for the integration of bio-molecular data have been proposed, several items remain open for current and future research. For instance, XML is mainly employed for the exchange format and in many cases the data management facilities (XSLT, XQuery, indexing structure,...) are not yet exploited. Besides this basic limitation, there are some other important issues in data integration which are not addressed by these systems:

• Data security and privacy. Data contains sensitive information about people that needs to be protected from unauthorized users. Specific approaches are required for biological data because they contain personal characteristics that can lead to the identification of a subject and their obfuscation can alter the experimental results.

• Evolution of data. Biological databases quickly change [91]: data formats, access methods and query interfaces are not stable over time, and even when elaborate database integration solutions are used, a significant amount of time is spent to address this issue.

• Efficiency. Approaches for the efficient evaluation of queries in a distributed and heterogeneous environment as well as approaches for collecting and normalize answers produced from independent sources should be developed.

• Approximation. The richness of data format and organization requires the development of systems that return approximate answers to an user query.

We have to remark that conflicts at physical and syntactic levels are almost solved exploiting XML technologies. However conflicts at the semantic layer are still an open issue for seamless biological data integration.

In the remainder of the section we present the main research initiatives that are currently devised to face these issues in the XML context.

### Towards more powerful representations of bio-entities

Despite the current standardization efforts, the Bioinformatics community still lacks of a standard exchange language and vocabulary for all the biological data. As shown along this paper, XML-like representations have been widely accepted to represent biological data. Additionally, several controlled vocabularies (e.g. thesauri) are now available to properly annotate these data. These vocabularies are usually expressed in the Open Biological Ontologies (OBO) [14], for example the Gene Ontology, the NCBI Taxonomy, the Cell Ontology, etc. The main drawbacks of these standards are that pure XML representations do not account for semantics, and that OBO ontologies are in most cases limited to simple taxonomies (i.e. informal *is-a *relationships).

The use of more expressive logics would give rise to more powerful and extensible ontologies so that biological concepts can be described not only with taxonomical relationships but also with logical descriptions (axioms). Consider, for example, the following pair of axioms

(1)

(2)

It can be derived that *GeneticInteraction *⊑ *Interaction*, that is, a new implicit *is-a *relationship is inferred from concept definitions. Notice that in this way, ontologies can be more compact and legible as concept descriptions are closer to natural language expressions.

To the best of our knowledge, BioPax is the only standard relying on an expressive ontology language. BioPax describes biological pathways and their components in the Ontology Web Language (OWL) [15]. In this way, specific pathway data can be classified according to the BioPax concepts by using a reasoner, as long as these data are represented as OWL *individuals*. It is worth mentioning that in OWL individuals do not need to be explicitly associated to a specific concept, but just to a proper description. This allows biologist to delegate the final classification to a reasoner. For example, taking into account the axioms 3 to 6 involving a set of individuals and the axioms 1 and 2, a reasoner is able to infer that *interaction*_1 is an individual of the concept *GeneticInteraction*.

(3)

(4)

(5)

(6)

Ontology-based data integration has been tested in systems like Ontofusion and Tambis previously presented. Ontofusion adopts a multiple ontology approach (e.g. one per source) whereas Tambis uses a unique global schema. Multiple ontology approaches are more scalable since they do not require a global ontology dependent of the data sources. However the implementation and integration is harder since the ontologies of each source should be integrated, that is, mappings between them have to be defined. This task may be rather difficult [92] if ontologies use different names or naming conventions to refer to their entities. Assuming that ontologies can be easily mapped (e.g. they use common vocabulary) semantic compatibility still arises as an open issue in ontology integration approaches. Ontologies to be integrated, and therefore the data sources, may contain conflicting descriptions which should be detected to perform a proper integration. This apparently disadvantage of the multiple ontology approach could also be seen as a strong, since ontologies could be exploited to detect those incompatibilities between data sources and then to repair/adapt them to make possible the integration. When integrating ontologies errors and incompatibilities manifest themselves as unintended logical consequences (e.g. unsatisfiable concepts or unintended subsumptions). In the literature several approaches can be found to detect and repair unintended logic consequences [93-95]. These techniques localize those sets of descriptions (i.e. axioms) which provoke the error (i.e. incompatibility).

Nevertheless, although the use of expressive ontologies seems to be a feasible solution to both the semantic representation of data sources and the classification of biological data, in practice, they are not being adopted as expected. The design of expressive ontologies requires strong skills in Description Logics (DL) [96], which are not familiar to biologists. That is why less expressive languages like OBO has become so popular among biologists.

### Open issues in service oriented architectures

The use of Web Services in Bioinformatics have been earlier analyzed in [97]. Some of the issues reported in this paper are being currently addressed, for example: the migration of HTML-based query forms to web service interfaces, the improvement of the discovery tools for biological web services (e.g. Semantic BioMOBY), and the overhead produced by XML when dealing with large biological data objects. However, there are some other issues that still remain open. Among them, we emphasize those related to data integration, namely:

• Web service architectures allow biologists to have several alternative sources for the information they request. In contrast, the selection of the proper sources will depend on criteria that are not usually found in these architectures, like the authority of the provider, the version of the data collection behind the service, etc. In this way, new metadata should be defined to guide users in the selection of the services they require for their tasks.

• Workflows also require some criteria and methods to select the services that potentially can comprise them [98]. These criteria must go beyond simple annotations of input/output parameters, because compositions can require more complex interactions between the involved services. For example, non-trivial data transformations may be required in order to connect two web services (i.e. Mediators). Additionally, we need the discovery of semantic mappings between WS data types to look for further potentially compatible services.

• Biological web services require an integrated data space consisting of just a few standard data formats, instead of the hundreds XML data types currently available. In this way, any data type used in a web service should be defined within a widely accepted semantic-based standard (e.g BioPax).

### Approximate retrieval of information

As earlier commented, data warehouse approaches allow a high degree of integration but at the cost of complying with a common database schema, which makes it difficult the inclusion of new data sources or the evolution of existing ones. Recently, several research works proposed to create XML data warehouses with data published in the Web (see [99] for a review). Basically, XML warehouses propose to store the XML data as it is without imposing any common schema. Afterwards, by applying clustering techniques and XML schema inference methods, the data warehouse provides the proper structures to support data exploration and analysis. However, these systems should face the high heterogeneity the stored XML data may present. Unfortunately, well-known XML tools like XPath and XQuery are not appropriate in this context, because they assume a well-defined schema.

Current approaches to handle highly heterogeneous XML collections are based on both approximate query processing [100,101] and multi-similarity systems [102]. The former consists of defining a relaxed query (pattern) in order to retrieve a list of similar XML documents (fragments). The latter ones provide multiple notions of similarity simultaneously in order to account for the heterogeneity of the data contained in the stored XML documents. The ArHex system [101] combines both methods in order to provide an extensible framework where users can adjust their similarity measures to the collection complexity. Such a framework could be used as the basis for defining novel exploration and analysis tools over highly heterogeneous biological data sets.

### Evolution of data

The rapid development of technologies leads to quickly change both biological data and applications working with such data.

For what concerns data, different problems should be faced. The introduction of new versions of data structures already developed leads to the problem of their management and also to determine the version on which queries should be evaluated. The evolution of data structures may imply the elimination of the old versions of data, but it introduces the issue of modifying existing instances in order to adhere to the evolved structures.

For what concern applications, the evolution of data structures requires to update the applications working on them in order to work properly with the different versions as well as the evolved structures. Moreover, mapping among schemas of two sources, when one of the two is modified, needs to be adapted.

The representation of biological data in the XML format can introduce further issues when modifying the schema (either represented through a DTD or a XML Schema). Specifically, the evolution of a schema may lead to revalidate documents already developed according to the old schema to check whether they are still valid for the new schema and, whenever they are no longer valid, to adapt the documents to the new schema. In [103], the X-evolution framework has been presented to address the issue of XML schema evolution. The authors propose both graphical and query-based approaches for the specification of schema modification and for adapting the documents to the new schema. Nevertheless, more specific approaches adapted to biological data should be addressed.

Schema modifications also impact on applications, queries, and mappings between schemas. The impact of schema evolution on queries and mappings has been investigated ([104-106]). The issue of automatically extending applications working on the original schema when this has evolved has not been addressed in the context of XML.

Last, but not least, another issue to be faced is ontology evolution; that is, the issue of modifying an ontology in response to a certain change in the domain or its conceptualization. The issues of ontology mapping, alignment, and evolution and their consequences on ontology instances should be addressed in the highly evolving context of biological data ([107-109]).

### Security and data privacy

The integration and management of heterogeneous data sources into a huge and organized data repository supports the scientists in making and proving the validity of their theories but it also produces as a side-effect the opportunity for a malicious user to access to or to make a prediction about relevant sensitive data. As an example, in healthcare domain a malicious user may be interested in patient genomic information in order to predict its current and future health status.

The degree of relevance of data and the kind of countermeasures to adopt in order to react against a malicious attack depend on several different aspects mainly based on the characteristics of the context to be considered and on the type of the attack.

Several approaches have been recently proposed to increase privacy and security in different context [110-114]. Access control, authentication, policy specification and enforcing techniques [115-117] are used to filter the requests to the sensitive resources so that the access requests coming from unauthorized parties be discarded and data be accessible only by users according to the enforced security policy. On the other hand, data obfuscation and data hiding techniques [118-120] are used to preserve privacy and security in presence of data mining techniques and they are based on the idea to distort or encrypt confidential data so that relevant information can not be easily retrieved.

When the security level increases, by adopting different security techniques coexisting together, the data sharing level decreases. Indeed, data are not publicly available but accessible only by those holding the required security credentials. A right tuning of these levels is desirable in order to satisfy both the security requirements and the data sharing demand.

## Conclusion

In this paper we pointed out the main current technologies that can be exploited for the integration and management of biological data through XML. We outlined the proposals for the representation of biological data in XML and discussed new interesting approaches that have been emerging in the last few years. We can conclude that XML has succeeded as the syntactic glue for biological data sources. Nevertheless, XML-based approaches produced a great variety of data formats, which makes it difficult to effectively integrate them. The adoption of a few semantic-rich standard formats is urgent to achieve a seamlessly integration of the current biological resources.

## Competing interests

The authors declare that they have no competing interests.

## Authors' contributions

The group of work is formed by Italian and Spanish teams. The idea and structure of this work has been proposed by MM. The Italian team was coordinated by MM, whereas the Spanish team was coordinated by EJR and RB. GV took care of the biological data types. MM, EJR, and PP presented the approaches for the XML representation of biological data. RB, EJR, IS, MM, PP, and DM worked on the data integration issues and approaches within the biological context. All authors jointly proposed the future work, read, and approved the final manuscript.
